# Toward a Neurobiologically Plausible Model of Language-Related, Negative Event-Related Potentials

**DOI:** 10.3389/fpsyg.2019.00298

**Published:** 2019-02-21

**Authors:** Ina Bornkessel-Schlesewsky, Matthias Schlesewsky

**Affiliations:** ^1^Centre for Cognitive and Systems Neuroscience, University of South Australia, Adelaide, SA, Australia; ^2^School of Psychology, Social Work and Social Policy, University of South Australia, Adelaide, SA, Australia

**Keywords:** neurobiology of language, event-related potentials, N400, LAN, MMN, P300, predictive coding, language comprehension

## Abstract

Language-related event-related potential (ERP) components such as the N400 have traditionally been associated with linguistic or cognitive functional interpretations. By contrast, it has been considerably more difficult to relate these components to neurobiologically grounded accounts of language. Here, we propose a theoretical framework based on a predictive coding architecture, within which negative language-related ERP components such as the N400 can be accounted for in a neurobiologically plausible manner. Specifically, we posit that the amplitude of negative language-related ERP components reflects precision-weighted prediction error signals, i.e., prediction errors weighted by the relevance of the information source leading to the error. From this perspective, precision has a direct link to cue validity in a particular language and, thereby, to relevance of individual linguistic features for internal model updating. We view components such as the N400 and LAN as members of a family with similar functional characteristics and suggest that latency and topography differences between these components reflect the locus of prediction errors and model updating within a hierarchically organized cortical predictive coding architecture. This account has the potential to unify findings from the full range of the N400 literature, including word-level, sentence-, and discourse-level results as well as cross-linguistic differences.

## Introduction: Linking Event-Related Potentials to the Neurobiology of Language via Predictive Coding

Event-related potentials (ERPs) have been central to theory-building in psycholinguistics and the cognitive neuroscience of language over the past decades. By contrast, the ability of ERP research to contribute to the emerging field of the neurobiology of language has proved more controversial (Small, 2008; Small et al., 2011). The neurobiology of language is a subfield of human neuroscience, with an explicit focus on the neural mechanisms underlying language (Small et al., 2011). Language-related ERPs, however, are often endowed with functional interpretations that are strongly linguistic or cognitive in nature, thus considerably limiting the scope of conclusions that can be drawn about neural mechanisms. Perhaps the clearest example of this is the long-standing debate about whether different linguistic subdomains (e.g., syntax, semantics) are associated with dissociable ERP components (e.g., LAN, P600 vs. N400) (For accounts in favor of this perspective, see for example Osterhout and Nicol, 1999; Friederici, 2002; Hagoort, 2005; for arguments against, e.g., Sassenhagen et al., 2014; Bornkessel-Schlesewsky et al., 2016). More general accounts are typically cognitive in nature, positing functions such as access to semantic memory for the N400 (Kutas and Federmeier, 2000, 2011) or conflict monitoring for the P600 (Kolk et al., 2003; van de Meerendonk et al., 2009), to name just two examples. While functional interpretations of this type are suited to informing linguistic or cognitive theories, the gap to neural mechanisms and, hence, the neurobiology of language is readily apparent.

Conversely, more general developments in cognitive neuroscience suggest that ERP-based research can indeed inform theory-building in a deeply neurobiological manner. The mismatch negativity (MMN) is a case in point, having featured prominently in studies on predictive coding and free energy minimization in the brain (e.g., Friston, 2005; Garrido et al., 2009; Moran et al., 2014). In addition to providing a possible unified theory of neural information processing across a wide range of different domains (for review, see Friston, 2010), predictive coding has been linked intimately to neurobiology via assumptions about cortical organization down to the level of cortical microcircuits (Bastos et al., 2012).

###  Outline of the Hypothesis and Theory to be Put Forward Here

Here, we propose that the insights gained in the context of the MMN and predictive coding can be fruitfully applied to understanding both the functional role and the neurobiology of the N400, as well as that of other language-related negative ERP components (e.g., the LAN). Specifically, we argue that N400 amplitude reflects precision-weighted prediction error signals, with high precision essentially amounting to conditions of low uncertainty regarding the role of a particular information source (see below for a more detailed exposition). The antecendent conditions of N400 effects are thus very similar to those of the MMN, and we therefore posit that the two components show shared characteristics as part of a functionally and neurobiologically related family of negative ERP components. Latency and topography differences between different members of the family can be attributed to the nature and complexity of the information being processed (e.g., single tones vs. connected speech in context).

On the basis of the hypothesized familial similarity between the N400 and the MMN, we put forward a new theory on the neurobiology of the N400. This proposal draws on the extensive literature linking the MMN to predictive coding and the empirical and theoretical progress that has been made on the neurobiological grounding of predictive coding architectures. We further suggest that this general framework also applies to other language-related negativities and that it contrasts with the functional role—and neurobiology—of language-related positivities.

###  Overview

The remainder of the paper is structured as follows. We begin by briefly reviewing the—much discussed and somewhat controversial—status of prediction in language comprehension, focusing specifically on the role that the N400 literature has played in informing this debate. Subsequently, we introduce the predictive coding framework, arguing that its application in the context of the N400-prediction debate has three advantages: (a) it renders a number of controversies on prediction and the N400 moot by showing how apparently competing perspectives can be integrated within a single architecture; (b) it allows for architectural and mechanistic assumptions about language processing to be derived within a more general framework for information processing in the human brain; (c) its neurobiological bases have been studied extensively. We then go on to describe in more detail how the cognitive and neurobiological assumptions of predictive coding have been applied in the context of a model component, the MMN, before motivating how they can be extended to negative language-related ERP components such as the N400. Language-related positivities, by contrast, are posited to have distinct functional and neurobiological characteristics. The final sections of the paper discuss the present account in relation to existing interpretations of the N400 and other relevant ERP components, concluding with an outlook and testable hypotheses.

It is important to note that this paper describes a new, neurobiologically grounded perspective on the N400 and other language-related negativities as recorded using scalp EEG. Its primary purpose is to describe this new account and the testable hypotheses arising from it, and it thus does not seek to provide an exhaustive review of the existing literature on the N400 or other language-related ERP components.

## Prediction in Language and the N400: Discussions and Controversies

The notion of prediction has played a prominent, but controversial role in the psycholinguistic and neurolinguistic literature. We will provide a short overview of this debate, focusing specifically on the role that the N400 has played in this context (for more comprehensive reviews, see Van Petten and Luka, 2012; DeLong et al., 2014)^1^.

In light of the well-established observation that N400 amplitude is inversely correlated with stimulus predictability^2^ (see Kutas and Federmeier, 2011, for a review), the idea that the N400 reflects prediction appears highly plausible at a first glance. However, findings of this type do not constitute conclusive evidence for prediction, as they are also compatible with varying integration costs for the current input item vis à vis the context (see Van Petten and Luka, 2012, for detailed discussion).

Additional discussion has focused on what is, in fact, meant by prediction. In this context, “active” prediction of a single upcoming word is often contrasted with “passive” anticipation of upcoming input, with the latter typically conceptualized in terms of the preactivation of particular stimulus features (cf. Kutas and Federmeier, 2000; Van Petten and Luka, 2012; DeLong et al., 2014). Arguments in favor of preactivation—and against prediction “proper”—have been advanced on the basis of several properties of the N400:

- N400 amplitude reduction for a target word appears to be feature-based rather than requiring full identity. This is evidenced by a range of studies that observed reduced N400 amplitudes for *unexpected* continuations sharing certain properties with the expected continuation. In the first study of this type, Federmeier and Kutas (1999) demonstrated this for category overlap with the expected continuation (e.g., “‘Checkmate,’ Rosaline announced with glee. She was getting to be really good at chess/monopoly/football”). N400 amplitude was largest for the between-category violation (“football”), intermediate for the within-category violation (“monopoly”), and smallest for the expected continuation (“chess”). Related effects have since been observed for orthographic similarity to the expected continuation (Laszlo and Federmeier, 2009)^3^, similar sensorimotor properties (Amsel et al., 2015), and association with the event under discussion independent of associations with individual words in the context (Metusalem et al., 2012).
- While there is widespread evidence for an N400 amplitude reduction for predictable words (or words related to predictable words), there is less support for N400 amplitude increases reflecting prediction mismatches. In other words, the N400 appears to mirror facilitation rather than penalties or processing costs arising from failed prediction.

Several studies have attempted to circumvent the problems arising from the attempt to measure prediction on a critical word—due to the general difficulty in separating prediction and integration at this position as discussed above—by measuring at a position preceding the critical word. The most prominent study of this type was conducted by DeLong et al. (2005), who presented sentences such as “The day was breezy so the boy went outside to fly *a/an* …” and observed an N400 for “an” vs. “a.” Here, “a” but not “an” is compatible with the expected continuation “kite,” while both are equally congruent to the preceding context. As this renders the N400 effect at the article difficult to explain via integration, the findings by DeLong et al. (2005) were long regarded as seminal evidence for prediction effects in the N400. Moreover, the data suggest that comprehenders not only predict word meanings, but that they also preactivate associated form-based properties. More recently, however, a controversy has arisen regarding this result, as a large-scale (*N* = 334) study failed to replicate DeLong et al. (2005)'s article N400 effect (Nieuwland et al., 2018). Nieuwland et al. (2018) thus conclude: “[o]ur results do not support the view that readers routinely pre-activate the phonological form of predictable words” (p.1).

However, a number of other studies in languages other than English support the fact that prediction mismatches prior to critical stimulus onset can give rise to N400 effects. Wicha et al. (2004) observed an N400 at the position of a determiner preceding a critical noun in Spanish, when the gender of the determiner did not match that of the predicted noun. Szewczyk and Schriefers (2013) reported an N400 for prenominal adjectives in Polish, when they did not belong to the animacy category matching that of the predicted noun. Finally, in a study on German sign language, Hosemann et al. (2013) found N400 effects time-locked to the handshape change preceding critical sign onset. Handshape change preceded sign onset by a mean of approximately 300 ms and was followed by a transition phase to the handshape for the target sign. Crucially, even though the transition phase in and of itself is not associated with a specific sign and does not bear any meaning, N400 amplitudes increased when the transition phase was not compatible with the predicted target sign. This result thus provides strong coverging support for prediction-based preactivation of form features prior to critical word onset.

In summary, we interpret the literature as having demonstrated prediction effects in the N400, but we also share the perspective of DeLong et al. (2014), who state that “[W]e feel obliged to point out that language processing is unlikely to proceed in either a strictly anticipatory (context exerting its influence prior to the receipt of target input) or strictly integrative (context exerting its influence only following receipt of target input) manner. In other words, under various circumstances, sentence comprehension likely arises through some combination of predictive and integrative mechanisms” (DeLong et al., 2014, p. 632) (For similar suggestions, see e.g., Federmeier, 2007; Lotze et al., 2011; Tune et al., 2014).

## Enter Predictive Coding

Re-examining the debate on prediction in language and the N400 from the perspective of predictive coding has at least three advantages. Firstly, it allows us to consider predictive processing in the context of a more general framework for how the human brain processes information. This constitutes a parsimonious approach to prediction in language: unless there is compelling evidence to suggest otherwise, it is appealing to assume that language behaves similarly to other cognitive domains in regard to prediction of upcoming information. Secondly, predictive coding provides a framework within which anticipatory (top-down) and integrative (bottom-up) processes naturally interact. In our view, it thus renders some of the debates on prediction in language processing moot, by demonstrating how both sides of an assumed dichotomy can co-exist within a single, independently motivated architecture (cf. the observation by DeLong et al., 2014, in the preceding section). Thirdly, the neurobiological underpinnings of a predictive coding architecture have been examined extensively, thereby opening up new possibilities for deepening our understanding of the neurobiology of language.

The first of these two advantages will be covered in the following subsection, which provides a brief introduction to predictive coding. We subsequently discuss the neurobiological assumptions underlying current predictive coding theories.

###  A Necessary Interplay Between Top-Down and Bottom-Up Processes

The predictive coding framework is built on the basic assumption that the brain actively constructs explanations for the causes of its sensory inputs (e.g., Friston, 2005, 2010). To achieve this, it maintains an internal generative model of the world, which maps (hidden) causes to sensory consequences. These hypothesized sensory consequences are constantly tested against actual sensory input, with action serving to selectively sample sensations for this purpose (cf. Friston et al., 2016). When there is a mismatch between the sensations generated by the internal model and those actually encountered, a prediction error ensues and the model is updated. The system thereby aims to minimize prediction error by minimizing the perceptual divergence between the model hypotheses and the true posterior distribution of the sensory input. Predictive coding and hypothesis testing is accomplished at multiple levels, from very precise, short timescales to much longer timescales with higher abstraction and less sensory precision (from percepts to concepts; see (Hohwy, 2013), for discussion). Prediction errors are propagated up the cortical hierarchy via feedforward connections, thus serving to update the predictive model at each level and determine priors for the next prediction. This amounts to a highly efficient coding scheme, as only the divergence between expected and actual inputs needs to be conveyed rather than the input itself (e.g., Attneave, 1954; Rao and Ballard, 1999).

Importantly, in this approach, sensory input and prediction error are equivalent to the extent to which the sensory input has not been predicted. Hence, the predictive coding literature often refers to prediction errors being “explained away.” In the words of Friston (2010, p. 130): “explaining away just means countering excitatory bottom-up inputs to a prediction error neuron with inhibitory synaptic inputs that are driven by top-down predictions […]. The reciprocal exchange of bottom-up prediction errors and top-down predictions proceeds until prediction error is minimized at all levels and conditional expectations are optimized.” Thus, from the perspective of predictive coding, we should not necessarily expect to observe a “special” (e.g., neurophysiological) error signal for an unpredicted input, but rather an attenuation of the signal accompanying a sensory input when that input is predicted. This observation, which will be discussed further in the context of the MMN below, neatly mirrors the generalization that N400 amplitude differences appear to result from an attenuation of the N400 for predicted stimuli rather than augmentation of the N400 for unpredicted stimuli. Recall from above that this has been used as an argument *against* prediction in the context of the N400. However, as demonstrated by predictive coding, N400 attenuation is, in fact, a perfectly possible accompaniment to prediction when prediction errors are equated with sensory signals that have not been “explained away.”

###  Neurobiological Grounding

In addition to its detailed mechanistic assumptions about the interplay between top-down predictions and sensory input, predictive coding architectures have been underpinned with detailed neurobiological design assumptions. At the macroscopic level (extrinsic connectivity), the observation that cortical areas are organized hierarchically (Felleman and Van Essen, 1991) provides an anatomical basis for the hierarchical organization of the predictive model. Moreover, Bastos et al. (2012) put forward a detailed account of how characteristics of a predictive coding architecture correspond to the components and intrinsic connectivity of canonical cortical microcircuits (for alternative architectonic assumptions, see Heilbron and Chait, 2017).

In brief, feedforward prediction error signals from lower cortical areas are received in the granular layer (L4). They are passed on to excitatory and inhibitory interneurons in supragranular (superficial) layers, which are thought to encode conditional expectations about hidden causes and states, respectively. This information—essentially corresponding to the updated model—is conveyed to pyramidal neurons in infragranular (deep) layers, encoding new predictions that are transmitted via feedback connections to lower cortical areas (and back to L4).

In addition, supragranular pyramidal cells serve to transmit prediction errors to higher cortical areas. While the ascending projections which serve to transmit prediction error signals up the cortical hierarchy are excitatory and can drive spiking activity in their target regions, the descending projections that convey predictions to lower cortical areas are likely inhibitory and both driving as well as modulating (Bastos et al., 2012). Finally, based on different oscillatory properties of the activity in superficial and deep layers, it has been proposed that feedforward (prediction-error-related) activity may be carried by higher frequencies, while feedback (prediction-related) activity is conveyed by lower frequencies (Maier et al., 2010).

**Figure 1:**
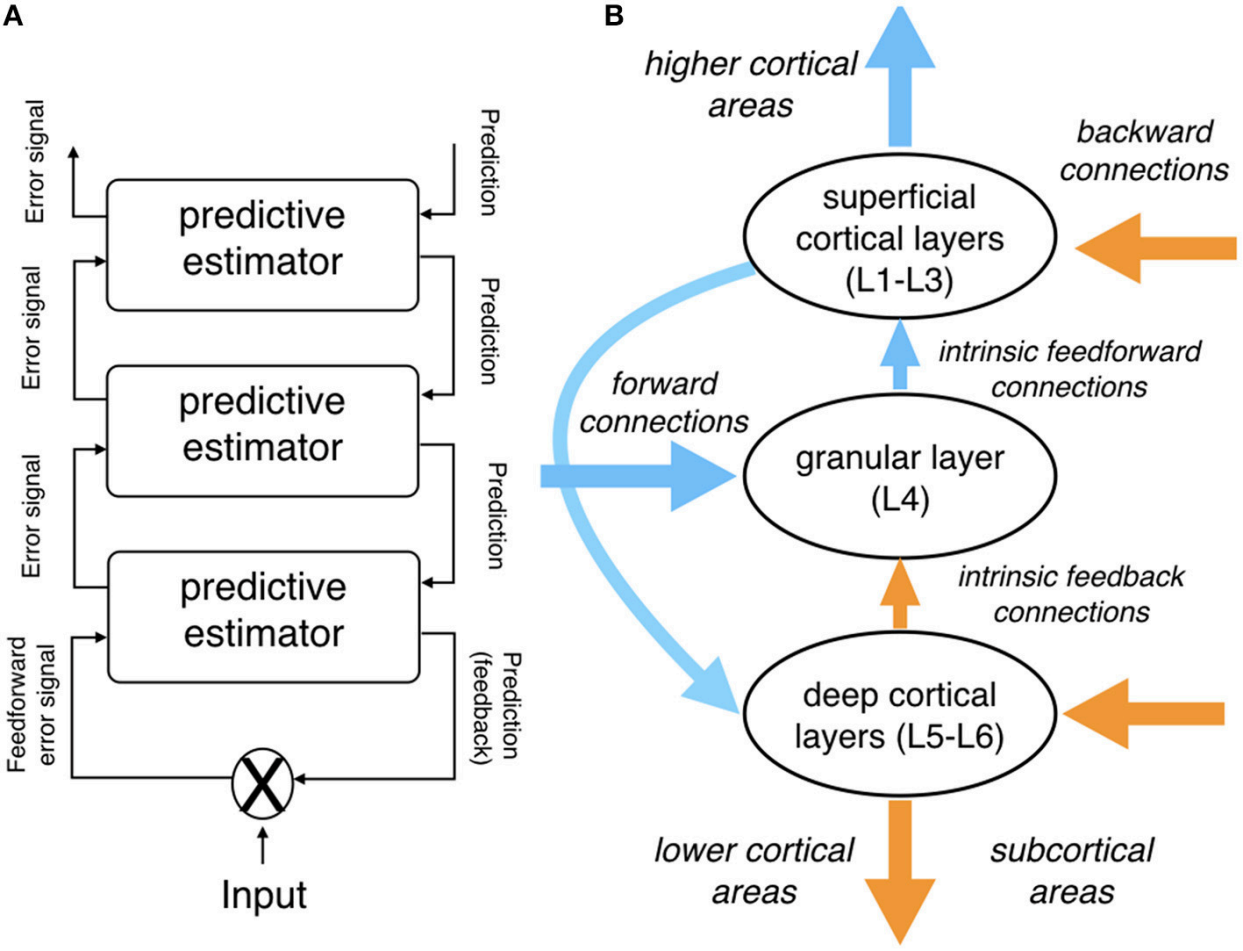
Schematic depiction of the predictive coding architecture. **(A)** Assumed hierarchical cortical organization of predictive estimators. Note how predictions are conveyed from higher to lower cortical areas via (anatomical) feedback connections, while prediction error signals are propagated from lower to higher cortical areas via feedforward connections. **(B)** Simplified depiction of a canonical microcircuit as assumed within the predictive coding literature; see main text for details. Note that, anatomically, each of the predictive estimator levels in **(A)** is thought to be instantiated by cortical microcircuitry as shown in **(B)**. **(A)** Was adapted with permission from Bornkessel-Schlesewsky et al. (2015); **(B)** was adapted and simplified from Bastos et al. (2012).

Figure 1 provides an overview of the predictive coding architecture and its neurobiological grounding.

## The Mismatch Negativity (MMN) as a Model Component

The cognitive and neurobiological assumptions in the previous section have been applied fruitfully in the context of the mismatch negativity (MMN, for a review, see Garrido et al., 2009), thus demonstrating the potential to investigate the neurobiology or predictive coding using scalp-recorded EEG. The MMN can be viewed as reflecting the prediction error—and, hence, the need for a model update—that arises when an unpredicted auditory stimulus is encountered. By contrast, the absence of MMN effects during the repeated presentation of a standard stimulus results from the improved predictability of the upcoming sensory input. In this case, prediction error-related activity in lower cortical areas is suppressed via the predictive feedback connections from higher areas; these serve to explain away the sensory input based on the assumptions of the generative model. As the model's ability to predict the input becomes more precise, less weight is assigned to bottom-up signals, thus reducing the post-synaptic sensitivity (gain) of prediction error units (Garrido et al., 2009).

By emphasizing the importance of integrating top-down predictions with bottom-up input, the predictive coding account of the MMN can explain a number of established findings, including that:

- MMN amplitudes are proportional to the perceptual divergence between the standard and deviant stimuli, reflecting the magnitude of the prediction error (Sams et al., 1985);
- MMN effects emerge via the reduction of ERP amplitudes for standards across a train of repeated presentations, reflecting the continual reduction in post-synaptic sensitivity for prediction error units with increasing predictive precision (Baldeweg et al., 2004)

It thereby unifies assumptions of previous competing accounts of the MMN based on model adaptation and adaptation to the standard stimulus, respectively (see Garrido et al., 2009, for detailed discussion).

The predictive coding account of the MMN is supported by studies using dynamic causal modeling (DCM), with model parameters reflecting the adjustment of connectivity to better reflect the input drawing on plastic changes in synaptic connections (e.g., Garrido et al., 2007). The explanatory power of such models has been demonstrated, for example, by their ability to account for age-related changes in ERP responses (Moran et al., 2014). Moran et al. (2014) assume that aging emphasizes model stability and reduces model complexity (avoidance of overfitting), thus leading to attenuation of Bayesian learning. In their words: “The implications for the neurobiology of aging are that—over the years—cortical message passing may become more efficient (providing accurate predictions with a less redundant or complex hierarchical model) and increasingly dominated by top-down predictions” (Moran et al., 2014, p. 2). This approach allows for the prediction of age from a DCM model of the MMN.

From this architecture, it is clear that predictive coding (i.e., neural message passing containing only the difference between the prediction and the actual input) is only part of the overall picture. There are other aspects of the architecture that could also drive the observed signals, including the model update itself, and the encoding of predictions. Moreover, prediction errors are weighted by precision / attention (Feldman and Friston, 2010; Kok et al., 2012). We shall return to this point below.

## The N400 as a Long-Latency MMN

In what follows, we will argue that the N400 is functionally similar to the MMN, with its longer latency and different topography reflecting the processing of more complex stimulus dimensions and longer temporal receptive windows, rather than a qualitatively different mechanism. We will subsequently argue that this approach can be extended to all language-related negativities. If this assumption holds, it would provide the basis for a deep neurobiological grounding of these observed effects, based on the predictive coding framework outlined in the preceding sections.

###  The Dual Importance of Prediction Errors and Prediction (Stimulus Probability)

We propose a functional link between the N400 and the MMN, based on two characteristics of the N400 that mirror properties of the MMN: (a) N400 amplitude reflects the degree to which an encountered stimulus diverges from an expected stimulus (Federmeier and Kutas, 1999), a property that has more recently been linked to the degree of surprisal associated with a word (Frank et al., 2015); and (b) N400 amplitude decreases proportionately to the degree of association between a word and the context in which it is encountered (Kutas and Hillyard, 1984). Property (a) has been discussed in terms of prediction and prediction errors (cf. Kuperberg and Jaeger, 2016, for discussion). However, recall from the N400 and prediction section that property (b) has led to the critique that N400 amplitude reductions for pre-activated stimuli may be driving the response rather than error-related amplitude increases (e.g., Van Petten and Luka, 2012). Indeed, in their review, Van Petten and Luka (2012) argue that there is little evidence in the literature to suggest that the N400 reflects prediction errors related to the deviation from a single predicted word. As argued above, however, while this pattern may be unexpected from the perspective of predictive processing in language in a traditional sense (i.e., active prediction of an individual word; incorrect predictions are costly), it is highly compatible with prediction as part of a predictive coding architecture.

**Figure 2:**
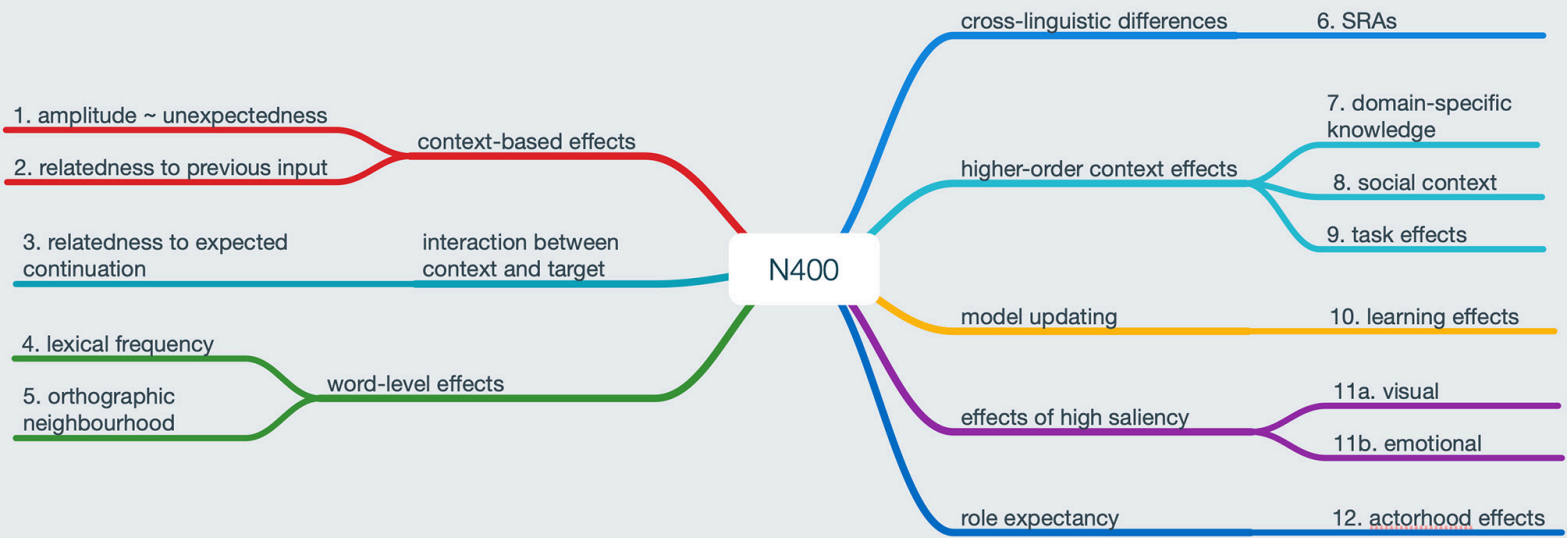
N400 modulations needing to be explained by a comprehensive account of the component. Left branches: “classical” N400 effects in context and at the word level (see, for example Kutas and Federmeier, 2011). Right-branches: additional effects at the sentence level and above. Effect 6 refers to cross-linguistic variability in the N400 response to semantic reversal anomalies (SRAs, see Bornkessel-Schlesewsky et al., 2011). Effects 7 through 9 and 10 refer to higher-order context effects and learning effects, respectively (see section “The Influence of Top-Down Modulations / Higher-Order Context Effects” in main text for references). Finally, effects 11a and b reflect N400 modulations through highly salient input stimuli (Lotze et al., 2011; Delaney-Busch and Kuperberg, 2013), while 12 is related to the actorhood potential of a noun phrase (Bornkessel-Schlesewsky and Schlesewsky, 2009b, 2013a; Frenzel et al., 2015).

Figure 2 summarizes the range of effects needing to be explained by a comprehensive account of the N400 (see the figure caption for further explanation and relevant references). We have already discussed types 1, 2, and 3 in the prediction and N400 section as well as above. Types 4 and 5 add an interesting additional perspective, as they can be observed at the single word level and thus, prima facie, cannot reflect prediction errors: N400 amplitude is negatively correlated with a word's frequency of occurrence (type 4); N400 amplitude also increases for pseudowords (i.e., word-like stimuli that respect the phonotactic constraints of a given language but are not existing words). Furthermore, for both existing and non-existing words, N400 amplitude is positively correlated with orthographic neighborhood density and frequency (type 5), i.e., higher N400 amplitudes for higher neighborhood density N and frequency (Laszlo and Federmeier, 2011). We assume that these effects can be explained from a predictive coding perspective via differences in a priori probability and uncertainty in stimulus categorization. Frequency effects may reflect global priors accrued over a lifetime of language experience. This also has the potential to explain why these effects become smaller or are even “overridden” by a sentence context when it is present (Van Petten and Kutas, 1990); for extended evidence from naturalistic story listening, see Alday et al. (2017). For neighborhood effects, we posit that N400 amplitude may reflect a prediction error (PE) effect somewhat akin to extra-classical receptive field effects in vision (Rao and Ballard, 1999). In other words, presentation of a (single) word not only leads to the processing of the word itself but also (at higher cortical levels) to the activation of words that are (semantically or orthographically) related. Feedback connections from these higher levels lead to a prediction-error-like response in the case of higher competition, i.e., in the case of a denser orthographic neighborhood, which increases the uncertainty of having correctly identified the word in question. In both cases, it appears plausible to assume that there is a prediction error component as well as a prediction component: the composite N400 effect results from the combination of an amplitude increase in the less expected case and an amplitude reduction in the more highly expected case.

This overall scenario can also account for N400 modulations of types 1 through 3, namely via amplitude increase for prediction errors and amplitude reduction for more strongly predicted stimuli.

###  N400 Effects Reflect Precision-Weighted Prediction Errors

Matters become more complicated when we consider scenarios in which there is a prediction error, but no N400 amplitude modulation. Semantic reversal anomalies (SRAs) in English and Dutch are a case in point (type 6 in Figure 2). SRAs are sentences involving a role reversal, i.e., sentences which would be plausible if subject and object (actor and undergoer) were to be exchanged. An example (from Kim and Osterhout, 2005) is given in (1); here, there is a good associative fit between “hearty meals” and “devour”, but “hearty meals” is in the wrong role in the current sentence context:

1. The hearty meals were *devoring* …

The absence of an N400 modulation for these sentences is typically discussed as reflecting a temporary semantic illusion (Kim and Osterhout, 2005; Kuperberg, 2016) or explained in terms of the high degree of lexical-semantic association between the critical word and the sentence context (association-based view of the N400). Thus, the most common current interpretations of these findings are:

1. There is no prediction error. This is based on the assumption that, in these sentence types, argument roles are not used to generate predictions (Chow et al., 2016b; Kuperberg, 2016) or that the generation of such predictions takes too long to be effective immediately (Chow et al., 2016a, 2018).
2. N400 amplitude doesn't reflect sentence-level predictions, but rather lexical-semantic associations / the benefits of semantic context (Brouwer et al., 2012; Van Petten and Luka, 2012).

However, both interpretations are problematic in view of additional results. Explanation (a) is not compatible with the finding that SRAs in English engender early anomaly effects in eye-tracking measures. Weiss et al. (2017) observed longer first-pass reading times for SRA sentences vs. controls at the position of the critical verb and the post-critical region. While these effects were modulated by the degree of semantic association, they were present in both high and low association conditions. This result provides strong evidence against the assumption that SRA-type sentences do not engender prediction errors, irrespective of the explanation for why a prediction was not generated. It is also incompatible with the notion of a temporary semantic illusion. Explanation (b) is contradicted by the cross-linguistic observation that SRAs do elicit N400 effects in languages other than English or Dutch, e.g., in German, Turkish, and Mandarin Chinese (Bornkessel-Schlesewsky et al., 2011)^4^, and that they can also elicit N400 effects in English under certain circumstances that cannot be explained with reference to changes in lexical-semantic relatedness (Bourguignon et al., 2012). We have previously argued that this overall pattern is explained most parsimoniously with reference to the types of features that are weighted most strongly for sentence interpretation: while English and Dutch rely strongly on word order cues for interpretation, German, Turkish, and Mandarin Chinese all have a flexible word order and draw more strongly on bottom-up cues such as case-marking and animacy.

**Figure 3:**
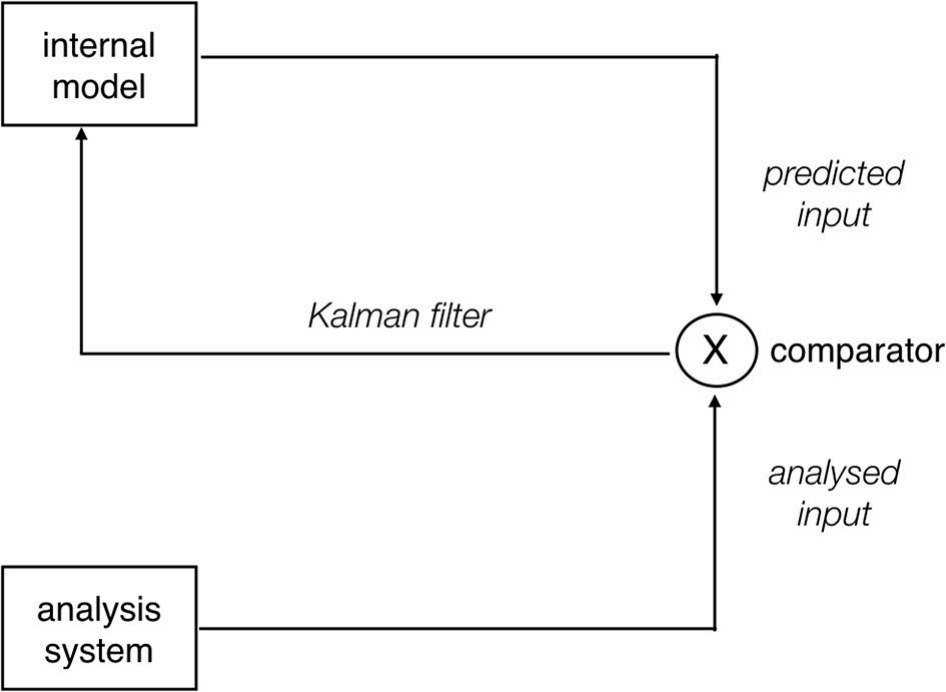
Schematic depiction of a predictive estimator allowing for precision weighting of prediction error signals. The prediction generated by the internal model is compared against the incoming input (“comparator”). If there is a mismatch between the two, a prediction error results. The degree to which this prediction error is used to update the model, however, depends on the precision of the error signal (see main text). This can be operationalized via a Kalman filter (Grush, 2004), which informally speaking determines to what extent the existing model is “trusted” over the current prediction error or vice versa. Modulations of update gain are also important in the context of noise, which can be present both in the context of the input and in the context of the model. The architecture shown here can be considered an elaboration of the predictive estimators shown in Figure 1A. Neurobiologically, precision-weighting of prediction error signals is thought to be implemented by modulations of the postsynaptic gain of pyramidal cells in superficial cortical layers, which serve to propagate the error signal to higher cortical areas; cf. top of Figure 1B.

We can thus conclude that, while SRAs induce prediction errors, these are not always reflected in N400 amplitude modulations. On the basis of this observation, we posit that, rather than reflecting prediction error-related activity *per se*, prediction-related N400 amplitude modulations reflect updates of the internal generative model. This explains the cross-linguistic findings on SRAs as follows. SRAs induce prediction errors in all languages. However, the effects of the mismatch-inducing linguistic feature differ from language to language. Consider animacy as the prime example of a mismatch-inducing feature in SRAs: the relevance of this feature for sentence-level interpretation is very low in English (as it is always overridden by other features such as word order), but higher in German or Turkish and very high in Mandarin Chinese. In other words, animacy has higher cue validity in these languages, as defined within the scope of the Competition Model (MacWhinney et al., 1984; Li et al., 1993): a cue is highly valid when it is both high in applicability (i.e., it is available when needed) and high in reliability (i.e., it is neither ambiguous nor misleading). We propose that cue validity, as a measure of how important particular cues are for interpretation in a given language, determines the *precision* of a prediction error induced by that cue during sentence processing. In the predictive coding literature, it is assumed that the feedforward error signals propagated up the cortical hierarchy are weighted by precision (Bastos et al., 2012), which is defined as the inverse of variance (Feldman and Friston, 2010; Kok et al., 2012). The notion of precision is thus directly related to the notion of cue reliability, i.e., the degree of variance in the form-to-meaning mapping for an individual cue in sentence interpretation. This proposal accords with the intuitive idea that a prediction error induced by a cue that is typically highly relevant for sentence interpretation in a given language should have a more substantial impact on model updating than a prediction error induced by a cue that is typically of low relevance for interpretation. Neurobiologically, this can be modeled by changes in the postsynaptic gain of the pyramidal cells in superficial cortical layers that encode prediction errors and propagate these to higher cortical areas (Bastos et al., 2012). Figure 3 summarizes the assumed mechanism underlying the precision-weighting of prediction error signals.

Further converging support for this proposal stems from the observation that the relevance of a cue for sentence interpretation may change depending on the circumstances in which it is encountered. For example, Bornkessel-Schlesewsky et al. (2011) reported that SRAs in Icelandic engender either an “English-type” ERP response (i.e., no N400 effect) or a “German-type” ERP response (i.e., an N400 effect) depending on the sentence type. Constructions requiring word-order-based argument interpretation (as in English) showed the English-type ERP pattern, while constructions requiring case-based argument interpretation (as in German and Turkish) showed the German/Turkish-type ERP pattern. Similar construction-based variability in the ERP-response to SRAs has, in fact, been observed in English: Bourguignon et al. (2012) compared typical SRA sentences involving action verbs with SRAs including experiencer verbs. For the experiencer constructions, they observed an N400 effect for SRAs in comparison to plausible controls, as well as a late positivity; SRAs with action verbs, by contrast, only engendered a late positivity—as in previous studies. We assume that this reflects the change in cue relevance for experiencer verbs: across the languages of the world, non-default verb classes such as experiencer verbs go hand-in-hand with different morphosyntactic properties in comparison to default (action) verbs (for empirical evidence from quantitative typology, see Bickel et al., 2014). English is no different: experiencer verbs can counteract the typical, word-order-based Agent-Action-Object template (Bever, 1970) by allowing the Causer to be the second argument (e.g., *Mary fears the dark*) and we assume that this increases the relevance of bottom-up (non-word-order) cues for sentence interpretation.

Modulations via the contextual relevance of stimulus features are another characteristic that the N400 shares with the MMN. MMN effects to speech sounds (vowels and syllables) are known to be modulated by phonemic status, i.e., MMN effects are induced based on whether two vowel sounds, for example, correspond to the same phoneme category in the listener's native language (or a non-native language in which they are highly proficient). Under these circumstances, the MMN is sensitive to linguistic representations rather than to acoustic similarity—as is the case in auditory oddball experiments involving tones rather than speech sounds (see Christmann et al., 2014, for recent findings using speech sounds and complexity-matched non-speech sounds; and Näätänen et al., 2007, for a review). Thus, the relevance of particular stimulus features for the MMN varies depending on the experimental context: if the stimuli can be mapped to existing linguistic categories, language-specific linguistic features play a critical role; by contrast, if the auditory input does not correspond to pre-existing categories, its acoustic parameters (e.g., frequency) are of primary relevance. Moreover, the MMN has also been interpreted as reflecting precision-weighted prediction errors on the basis of biases introduced by higher-order sound sequences (Todd et al., 2014).

###  The Influence of Top-Down Modulations / Higher Order Context Effects

It has been known for some time that the N400 is sensitive to a range of influences beyond the current sentence context (Hagoort and van Berkum, 2007). Many of these can be explained relatively straightforwardly within the current framework by assuming that contextual information shapes the current predictive model, irrespective of whether it is part of the current sentence context or not. Here, we discuss a selection of recent findings which attest to the exquisite sensitivity of the N400 to subtle manipulations of the top-down predictive environment.

Troyer and Kutas (2018) examined the role of an individual's domain-specific knowledge in eliciting N400 effects by having participants read sentences from the domain of the Harry Potter (HP) novels. Endings that were more or less contextually supported within the HP universe showed N400 effects that were graded according to individual participants' HP knowledge. For non-HP-related sentence endings, by contrast, there was a general effect of contextual support. This result (type 7 in Figure 2) is exactly in line with the assumptions of predictive coding: internal generative (predictive) models are shaped substantially by individual expertise.

Further results (Rueschemeyer et al., 2015) attest to the sensitivity of the N400 to the social context in which a linguistic utterance occurs (the “social-N400 effect”; type 8 in Figure 2). In this study, participants read sentences following an auditorily presented context sentence, and performed the experiment either on their own or in the presence of a confederate, who saw the target sentences but did not hear the context sentences. Target sentence endings were either: plausible independent of the context sentence; implausible independent of the context sentence; plausible or implausible depending on the context. Participants who were in the alone-group had the task of judging whether they had understood the sentence, while participants in the joint-group were asked to judge (a) whether the confederate understood the sentence, and (b) whether they themselves understood the sentence. Participants showed an N400 effect for the context condition in comparison to the plausible condition only in the presence of a confederate, thus showing that they adapt their predictive model to the social context at hand. Jouravlev et al. (2019) showed that this is also possible without explicit task instructions. These findings are again fully compatible with a predictive coding perspective, as predictive coding has previously been argued to extend to social interactions including theory of mind (Koster-Hale and Saxe, 2013). Once again, we view this as attesting to the flexibility of predictive model adaptation depending on prior knowledge and the current context (for related results involving the social status of a speaker, see Bornkessel-Schlesewsky et al., 2013).

Similar considerations hold for modulations of the N400 by task environment (type 9 in Figure 2). As shown by Chwilla et al. (1995), N400 priming effects elicited by word pairs in the context of a lexical decision task were not present in the context of a physical task (case judgement). This demonstrates that predictive models are adapted to best fit the current environment: in the absence of a broader context, prediction between the individual words of a word pair is strengthened through the intra-experimental context (i.e., the fact that, of the word pairs presented, some are semantically related). This knowledge, however, is only relevant when participants are performing a lexical decision task, and thus does not influence the predictive model in the context of a physical task. The scenario changes when the linguistic input contains intrinsic predictive properties, as is the case in the presence of a sentence or discourse context. Accordingly, N400 effects are observable under such conditions even in the absence of an explicit, meaning-probing task (see, for example, (Alday et al., 2017), for evidence from naturalistic story comprehension)^5^.

###  Model Flexibility / Learning

The findings on domain-specific knowledge discussed above suggest that individuals must be able to flexibly learn, i.e., adapt their predictive models to new environmental probabilities. This process can, apparently, be very rapid if the learning environment is sufficiently specific/informative. Borovsky et al. (2012) exposed participants to high and low constraint sentences (from Federmeier and Kutas, 1999, see the “Prediction and the N400” section). These involved either plausible/expected completions or were completed with an unknown pseudoword. Subsequently, participants were presented with a word-pair priming paradigm, comprising identical, related, and unrelated word pairs. Strikingly, N400 priming effects were induced by unknown word primes after only a single exposure to these novel words in context. However, this was only the case when the pseudoword had appeared in a high-constraint context. This suggests that learning/model adaptation can be very rapid, occurring with just one exposure. However, this is contingent on the evidence being strong enough during that single-shot exposure to warrant a model update—likely to avoid overfitting through unnecessary model adaptations to any unpredicted stimulus (cf. Moran et al., 2014).

## Extension to Other Language-Related Negativities

In the preceding section, we argued that the N400 shares crucial characteristics with the MMN and that—given the availability of detailed, neurobiologically grounded accounts of the MMN—viewing the two ERP components as part of the same family opens up potentially important new perspectives on the neurobiological mechanisms underlying the N400. Specifically, we have posited that both the MMN and the N400:

- reflect dual aspects of stimulus prediction (leading to an amplitude decrease for predicted stimuli) and prediction error (leading to an amplitude increase for stimuli that do not match an existing prediction);
- are sensitive to the contextual relevance of stimulus features, such that only currently relevant features give rise to a prediction-error-related response (precision-weighted prediction errors);

We assume that the N400 is thus essentially a long-latency MMN, with the latency difference reflecting the higher stimulus complexity in N400 paradigms. This mirrors the well-known latency shifts of the P300 in response to the complexity of stimulus analysis (cf. Donchin and Coles, 1988; Polich, 2007, among many others). An MMN-type response with a longer latency than the classic MMN—known as the late MMN or late discriminatory negativity (LDN)—has, in fact, also been reported in the literature. The late MMN manifests itself at fronto-central electrode sites, with a latency of approximately 300 to 600 ms, and occurs in response to complex auditory stimuli (e.g., syllables, words) as well as auditory rule extraction (e.g., Zachau et al., 2005). It has been proposed that these two ERP responses form part of a hierarchically organized predictive coding system, with the early MMN reflecting local regularities (predictions) and the late MMN reflecting higher-order regularities or predictions (Wacongne et al., 2011). From this perspective, functionally related negativities with differing latencies and topographies—reflecting different underlying neural generators—arise as a result of the hierarchical cortical system that is assumed to underlie perceptual inference and predictive coding in the human brain. In essence, we can conceive of this as a similar mechanism occurring throughout a distributed network, namely the matching of top-down prediction signals to bottom-up sensory/prediction error signals. Precision-weighted prediction error signals can arise in various loci throughout this network, as part of one sweep through the hierarchically organized system, and likely even in multiple loci at once for a complex stimulus such as language. Accordingly, these signals—though functionally equivalent—can manifest with different latencies and topographies in scalp-recorded EEG, depending on their point of elicitation within this complex system.

In addition to positing that the N400 forms part of this family of predictive coding-related negativities, we propose that an explanation along these lines can be extended to other language-related negativities such as the left-anterior negativity (LAN). The LAN, which has a similar latency to the N400, was traditionally associated with morphosyntactic violations such as violations of subject-verb agreement (e.g., Friederici, 2002). However, there is not a clear one-to-one mapping between the occurrence of a morphosyntactic processing problem and the occurrence of a LAN effect: sentences containing morphosyntactic violations or requiring a reanalysis (cued by a morphosyntactic information source, e.g., word order reanalysis on the basis of subject-verb agreement information) have been shown to engender N400 effects in a number of experiments; in other cases, morphosyntactic violations only elicited late positivity effects without a preceding LAN (or other negativity) (see Sassenhagen et al., 2014, for a summary of relevant findings).

We have previously argued that LAN effects may reflect morpheme-based expectations (Bornkessel-Schlesewsky and Schlesewsky, 2009a), i.e., prediction-error-related activity in the morphemic domain. From this perspective, the LAN could be afforded a similar functional interpretation to the MMN and the N400, with the topographical and latency differences between the three components reflecting differences in the input features that are relevant for engendering the prediction error (see also Bornkessel-Schlesewsky and Schlesewsky, 2013b). By extension, early LAN (ELAN) effects elicited by word category violations (e.g., Neville et al., 1991; Hahne and Friederici, 2002) could reflect prediction error effects in the context of unpredictable category sequences (but see Steinhauer and Drury, 2012, for a critical discussion of ELAN effects).

Note that our assumptions about the LAN are, at present, a conjecture. While they provide a parsimonious explanation for language-related negativities in general, they clearly require experimental validation. To date, there is mostly evidence to support a prediction-error-based enhancement of the LAN, rather than the dual aspects of prediction and prediction error (involving a clear prediction-related attenuation) as discussed above for the MMN and the N400. It is not clear whether this is due to the characteristics of the experimental designs which have been used to study the LAN and which may not have been suited to revealing prediction-related aspects, or whether it should be attributed to characteristics of the component itself. For example, while classic N400-eliciting designs such as variations of cloze probability offer a straightforward means of differentiating between highly predictable, neutral, and highly unpredictable continuations, this is not possible in the same way in typical morphosyntactic manipulations. Consider subject-verb agreement: here, the agreement features are either predicted or unpredicted (in the case of a violation), but—at least typically—there is no middle ground. This makes it difficult to disentangle prediction-related and prediction-error-related aspects of the effect. Also, there is no direct evidence as yet to support the idea that LAN effects, like N400 effects, reflect precision-weighted prediction error responses rather than prediction errors *per se*. However, though there is no compelling empirical evidence to date, the assumption that LAN effects also reflect precision-weighted prediction errors could have the potential to explain why morphosyntactic manipulations sometimes elicit LAN effects, while under other circumstances they do not (for a review, see Bornkessel-Schlesewsky and Schlesewsky, 2009a). This otherwise somewhat puzzling observation has already been discussed in the context of possible differences between different morphosyntactic agreement features (Nevins et al., 2007)—an idea that is clearly related to our proposal here.

In summary, we propose here that language-related negativities – including the N400, LAN and ELAN – form part of a functionally and neurobiologically unitary family of components which, like the MMN, index precision-weighted prediction errors. The latency and topography of the resulting negativity effects is assumed to reflect the locus of the prediction error within the overall, hierarchically organized cortical architecture (cf. Bornkessel-Schlesewsky and Schlesewsky, 2013b). To some extent, the latency of the resulting negativity effects is thus also expected to mirror the relative genesis of the prediction error within the overall cascade. We deliberately say “to some extent,” as hierarchical cortical organization does not imply a perfect feed-forward architecture. Rather, different levels of the hierarchy are typically connected bidirectionally; furthermore, connections can be long-range, i.e., “skip” individual levels (e.g,. Bar, 2003; Kravitz et al., 2013). Thus, the time-space-correspondence (TSC) of prediction error effects (Bornkessel-Schlesewsky and Schlesewsky, 2013b) can be assumed to be imperfect at best. Nevertheless, the existing literature provides at least some evidence for TSC in a restricted sense. As evidenced by MEG studies of sentence reading, word category violations engender early (M100) prediction error effects localized to visual cortex (Dikker et al., 2009). Moreover, these effects are modulated by form typicality (Dikker et al., 2010), i.e., they occur only for orthographically typical nouns (e.g., “the tastelessly *soda*” vs. “the tasteless *soda*”) but not for orthographically atypical nouns (e.g., “the cutely *infant*” vs. “the cute *infant*”). These results suggest that, when the predictive model is precise enough, the language comprehension system generates predictions right down to the level of expected sensory—in this case orthographic—inputs. In this case, prediction error effects can be observed “early”, both in terms of timing and in terms of the cortical hierarchy. Similar effects have been observed for semantically-based prediction effects (Dikker and Pylkkänen, 2011), thus attesting to the importance of the prediction itself rather than the information used to generate it.

## Relation to Language-Related Positivities

Our proposed approach to language-related negativities contrasts with our perspective on language-related positivity effects (e.g., the P600). As we have discussed in detail elsewhere (Sassenhagen et al., 2014; Sassenhagen and Bornkessel-Schlesewsky, 2015), we view these effects as part of the P300 family (see also e.g., Gunter et al., 1997; Coulson et al., 1998). Specifically, we follow Nieuwenhuis et al. (2005) in assuming that the P300 reflects a phasic release of noradrenaline by the locus coeruleus (LC), a brainstem nucleus, to motivationally significant stimuli (the “LC-P3 model”). This leads to a stronger neuronal reactivity (gain) to the stimuli in question, thereby optimizing the appropriate behavioral reaction. As shown by Sassenhagen et al. (2014) and Sassenhagen and Bornkessel-Schlesewsky (2015), P600 effects share a number of characteristics with the P300 as expected from the perspective of the LC-P3 model, including single-trial-based locking of P600 latency to behavioral outcomes (reaction time) and correlation with other physiological reactions tied to the (LC-initiated) orienting response (e.g., galvanic skin response, GSR). Thus, in comparison to language-related negativities, which are stimulus-locked, language-related positivities are response-locked, thereby reflecting their closer link to the behavioral consequences of a predictive coding response, rather than to predictive coding *per se*.

## Relation to Existing Theories

###  Comparison to Existing Accounts of the N400

The key difference between our proposal and existing accounts of the N400 is our assumption that the N400's prediction-error-related component reflects *precision-weighted* prediction errors, with precision (the inverse of variance) reflecting the relevance of a particular stimulus feature in a given language. At some levels, “relevance” may be immutable for a particular language—for example, in the case of the phonetic features that do or do not give rise to phonemic contrasts. In other cases, it may be more context-dependent: at the level of sentence interpretation, for example, we have argued that cue validity in the sense of the Competition Model (e.g., MacWhinney et al., 1984) can be used as a proxy for relevance/precision, but the importance of an individual cue such as case marking or word order can change depending on the construction being processed (see the above discussion of the data from Icelandic and English by (Bornkessel-Schlesewsky et al., 2011; Bourguignon et al., 2012), respectively). Precision-weighting of prediction errors has not previously been proposed in the context of the N400 or other language-related negativities (but see e.g., Todd et al., 2014, for the MMN).

Our account also differs from other current approaches to the functional significance of the N400 in assuming that the N400 is functionally equivalent to the MMN as well as to other language-related negativities. We hope to have shown how the assumption that the N400 reflects similar basic neural mechanisms as the MMN opens up a whole host of new possibilities for ERP-based language research on the neurobiology of language. These could build on the detailed neurobiological accounts of hierarchical predictive coding that have been put forward and linked to the MMN, including, for example, the proposed separability between units encoding predictions and those encoding prediction errors within canonical neural microcircuits (Bastos et al., 2012). Moreover, by not restricting our assumptions to semantic information—as is the case in most, if not all, competing approaches—we are able to derive non-semantic N400 effects, e.g., increased N400 amplitudes for disambiguation toward a non-preferred word order (e.g., Bornkessel et al., 2004; Haupt et al., 2008; Hörberg et al., 2013) and for case violations (e.g., Frisch and Schlesewsky, 2001)^6^.

Of course, our proposal also shares a number of commonalities with existing approaches to the N400. It shares the assumption of semantic memory-based accounts (e.g., Lau et al., 2008; Brouwer et al., 2012; Stroud and Phillips, 2012; Van Petten and Luka, 2012; Chow et al., 2016b) that N400 amplitudes are reduced for stimuli that fit into the current semantic context or have a high a priori probability in the absence of a context (e.g., high frequency words). By contrast, and in line with surprisal-based accounts (Frank et al., 2015; Kuperberg and Jaeger, 2016), we also assume that N400 amplitude reflects prediction errors—at least in part. In a recent proposal that is conceptually related to the update of an internal model via prediction error signals, Rabovsky et al. (2018) view the N400 as indexing the change in an implicit and probabilistic meaning representation that is elicited by an incoming stimulus. From this perspective, N400 amplitude reflects the magnitude of the update as simulated within a computational (neural network) model (cf. also Rabovsky and McRae, 2014, in which N400 effects are simulated as semantic netowrk errors). Thus, while entertaining related notions of a continuous model update with surprisal-based accounts and Rabovsky et al. (2018)'s computational model, our proposal differs in assuming a key role of precision weighting^7^. This additional assumption is necessary in order to explain the cross-linguistic and construction-based variability of N400 effects (cf. type 6 in Figure 2 and the section “N400 effects reflect precision-weighted prediction errors”).

Finally, like several other existing proposals (Kuperberg, 2016; Chow et al., 2018), we assume a processing architecture that generates and tests predictions at a number of different levels and, potentially, at differing timescales. In contrast to these other approaches, however, we view the different predictive levels as linked to the hierarchical organization of a cortical predictive coding architecture, within which predictions are generated and tested at each level. From our perspective, timescale differences go hand-in-hand with the increasing length of temporal receptive windows at increasingly higher levels of the hierarchy (Hasson et al., 2008; Lerner et al., 2011; Bornkessel-Schlesewsky et al., 2015; Kandylaki et al., 2016), and prediction/prediction-error effects may be elicited at any level of the hierarchy—likely at multiple levels in many cases^8^. The negative ERP responses reflecting predictive processing and its dual components of prediction and prediction error are assumed to show different latencies and topographies depending on the networks within the overall architecture in which they are generated (cf. Moran et al., 2014, for age-related changes in the connectivity of networks underlying the MMN; and Wacongne et al., 2011, for evidence of hierarchical differences in information processing and concomitant changes in the MMN)^9^.

###  Comparison to Other Existing Accounts of Language-Related ERP Components

Beyond the N400 literature, the assumption that language-related negative ERP responses reflect predictive processing at multiple, hierarchically organized levels also provides a link between the current approach and a body of work by Shtyrov, Pulvermüller and colleagues, in which they used MMN oddball paradigms to examine linguistic manipulations (e.g., Shtyrov and Pulvermüller, 2002; Pulvermüller and Shtyrov, 2003). The results of these studies revealed MMN modulations for different types of linguistic violations, including subject-verb agreement (Shtyrov and Pulvermüller, 2002; Pulvermüller and Shtyrov, 2003; Hasting et al., 2007), word category (Hasting et al., 2007) and semantic violations (Menning et al., 2005; Shtyrov and Pulvermüller, 2007), thus leading the authors to conclude that N400s and other late language-related effects are not informative with regard to the timecourse of language processing. Rather, all relevant processes (phonological, lexical, syntactic, and semantic) are thought to take place before 200 ms in a near-simultaneous, cascaded form (Pulvermüller et al., 2009), as reflected in the MMN findings. N400, LAN, and other later effects, by contrast, are assumed to be indicative of either a second stage of information processing or a “post-processing” stage. We agree with Pulvermüller, Shtyrov and colleagues that there are likely language-related effects that precede the N400, LAN, and other late components. This is to be expected within a hierarchically organized predictive processing architecture in which predictions are generated and tested at a number of different levels and timescales. However, rather than viewing later effects as reflecting secondary or post-processing stages, we would assume that they are merely reflections of predictive processing at higher hierarchical levels and, thereby, reflect processing at higher levels of complexity (cf. Simon, 1962). Given the organization of the hierarchical predictive coding architecture, in which prediction errors at lower levels are propagated up the hierarchy and thereby induce continuous model updating at various hierarchical levels, it appears highly likely that linguistic prediction errors at low levels will often be accompanied by higher-level prediction errors. For example, when predicting a particular word, there may first be a mismatch between the predicted speech sounds and those actually encountered, but this will necessarily also amount to a prediction mismatch with regard to the identity and meaning of the word—and possibly other aspects such as its word category as well. From this perspective, multiple effects at different timescales may reflect the overall operation of the predictive coding system and arise as a natural consequence of its hierarchical organization.

Note also that we do not assume that meaning is accessed at a single level within the hierarchically organized predictive coding architecture. Rather, it emerges as a result of the system's dynamics as a whole. In this regard, we agree with Kutas and Federmeier (2011), who stated in their review of 30 years of the N400 literature that “the meaning of a stimulus is not computed at a single point in time, but rather is something that emerges through time, with the activity measured in the N400 representing an important aspect of that emergent process, but not, certainly, the final state” (p. 642)—nor, we would add, necessarily the first state.

###  Comparison to Other, Broader Accounts of Event-Related Potential Components

We claim here that N400 effects and other language-related negativities reflect internal model updates via precision-weighted prediction error signals. Conceptually, this proposal appears rather similar to the context updating theory of the P300 (Donchin and Coles, 1988). While this is indeed the case, we view the current range of evidence as more strongly supporting the notion of model updating within the N400 (and other negativity effects) as opposed to the P300 (or language-related positivity effects). Two key arguments of the context updating approach were that P300 amplitude reflects subjective probability of a stimulus and that it is not strongly linked to behavior/task execution, as revealed by an absence of a correlation with reaction time (RT). As discussed in the “Relation to language-related positivities” section above, however, more recent findings indeed show that P300 effects are stongly RT-locked at the single trial level; this also holds for P600 effects in language and contrasts with the stimulus-locked nature of N400 effects (Sassenhagen et al., 2014; Sassenhagen and Bornkessel-Schlesewsky, 2015). Thus, there does appear to be a closer link between the P300 and behavioral consequences of a model update than to the (stimulus-driven) model update itself. This is in line with the assumptions of the LC-P3 model, in which the increase in neuronal gain to a salient stimulus that is accompanied by the P300 supports appropriate behavioral action execution. Moreover, it has become apparent that P300 effects can occur even when no model update is necessary, e.g., for self-relevant stimuli such as one's own name (see Brilmayer et al., 2018, for a brief review and an application to language processing). This supports the LC-P3's assumption that P300 effects reflect the motivational salience of a stimulus rather than its subjective probability.

Finally, note that the assumption that negative ERP responses such as the MMN and N400 may warrant a unified functional explanation has been put forward previously (Kotchoubey, 2006). In his approach, negativities are assumed to reflect sensory expectations (“questions”), while positivities such as the P300 are assumed to reflect feedback (“answers”) that either confirms or updates these expectations. Cortical information processing thus operates on the basis of this question—answer cycle, reflected in a general negativity—positivity ERP pattern. Applied to language comprehension, Kotchoubey (2006) posits that N400 and LAN effects reflect the building up of a “model of possible content” (p. 59) under conditions of high uncertainty. This leads to the scanning for further information by neural assemblies and, ultimately, a positive ERP component (e.g., a P600) when the crucial information is obtained. Thus, while the current approach concurs with (Kotchoubey, 2006) observation that “ERP components, thus construed, do not reflect the activity of specialized modules like syntax and semantics, but rather, the same basic ups and downs of cortical activity underlying the control of behavior, verbal behavior being a specific form thereof” (p. 59), the functional interpretation of negativite and positive ERP components offered here is quite different to his. Similar considerations apply in regard to Kotchoubey's neurobiological assumptions. Like the predictive coding approach (Friston, 2005; Bastos et al., 2012), he posits a functional separability between activity in superficial and deep cortical layers. Kotchoubey assumes, however, the formulation of expectations—as reflected in negative ERP responses—is associated with activity in superficial layers and feedforward connections, while feedback connections arising in deep layers are associated with expectation confirmation or updates (i.e., the basic anatomical assumptions are the inverse of those posited within the predictive coding framework).

## Outlook and Hypotheses

As noted at the beginning of this paper, our objective here was to put forward a new perspective on the functional interpretation of negative language-related ERP components and their neurobiological foundations. Our approach is motivated by recent advances regarding the neurobiology of perception and cognition—notably the notion of perceptual inference, as supported by a hierarchically organized, cortical predictive architecture—and a critical synthesis of the language-related ERP literature. Nevertheless, it remains, at present, a proposal that requires further concretization and empirical testing.

Perhaps most importantly, the notion of precision-based weighting of prediction errors needs to be operationalized further and quantified. It will be crucial to test quantitative hypotheses in this regard; otherwise, precision-weighting could be used in a relatively arbitrary manner to account for effects of varying amplitudes or absent effects that cannot otherwise be explained. We suggest that, in a first instance, cue validity in the sense of the Competition Model (MacWhinney et al., 1984) (or, possibly, as measured empirically via speaker judgements; Alday et al., 2015) could be used to quantify the precision of features related to sentence interpretation. The hypothesized impact of this precision weighting can be examined most directly in simple transitive sentences with default verb classes (action verbs) in order to avoid influences of contextually-induced modulations, which may prove more complex to quantify. The examination of annotated naturalistic stories could prove to be a key approach in this regard (Alday et al., 2017; see also Frank et al., 2015 for a related approach using visually presented sentences, and Brennan et al., 2012, for an approach using fMRI).

Finally, selected testable hypotheses arising from our approach are:

- Separable effects of prediction and prediction errors in language-related negativities. This follows from the assumption that predictions and prediction errors are encoded by different units. In addition to applying to ERPs, this hypothesis also relates to oscillatory brain activity. In line with the observation that feedforward, prediction-error-related activity is conveyed at high frequencies, while feedback, prediction-related activity is conveyed at lower frequencies (Bastos et al., 2012), we hypothesize that prediction and prediction-error-related frequency activity in language will show a comparable dissociation.
- Different levels of prediction tied to hierarchical predictive cycles. In the current approach, different levels of prediction are assumed to be directly tied to different levels of the hierarchical cortical predictive coding architecture. The temporal receptive windows (TRW) associated with these different levels (cf. Hasson et al., 2008; Lerner et al., 2011; Bornkessel-Schlesewsky et al., 2015; Kandylaki et al., 2016) provide an upper bound on the length of individual predictive cycles. TRW length can be estimated empirically by examining the entrainment between oscillatory EEG/MEG activity and linguistic units in the speech input (Ding et al., 2016).

## Author's Note

This manuscript was written in RMarkdown using the R package *papaja* (Aust and Barth, 2018).

## Author Contributions

IB-S and MS jointly conceived the theory and hypotheses presented here and wrote the paper.

### Conflict of Interest Statement

The authors declare that the research was conducted in the absence of any commercial or financial relationships that could be construed as a potential conflict of interest.

## Footnotes

^1^For discussion of ERP components other than the N400 that have been linked to prediction, see Van Petten and Luka (2012) and Nieuwland (2019). These are beyond the scope of the present paper.

^2^Predictability is typically measured via the cloze task, in which an independent group of participants is asked to provide a continuation for a sentence (or text) fragment. For a more detailed discussion of cloze probability and the N400, see Kutas and Federmeier (2011). Some recent approaches have also attempted to quantify predictability in terms of *surprisal* (a word's negative log-probability), an information-theoretic measure indexing the amount of information a word conveys (cf. Levy, 2008). See Frank et al. (2015) and Kuperberg (2016), for discussion of N400 amplitude modulations in terms of surprisal.

^3^Note, however, that there has been a discussion about the timing of the orthographic relatedness effect (see Ito et al., 2016; DeLong et al., 2018).

^4^Recent findings by Chow et al. (2018) appear to contradict the claim that SRAs elicit N400 effects in Mandarin—at least when the verb is encountered immediately after the arguments. However, this finding in fact replicates the pattern of results reported for the type of construction in question (bă-constructions) by Bornkessel-Schlesewsky et al. (2011). In our previous paper, we suggested that the absence of N400 effects for SRAs in bă-constructions—together with the presence of an N400 effect in bèi-constructions—might be due to the availability of an alternative (relative clause) analysis in the bă-sentences. This consideration also applies to Chow et al.'s (2018) results.

^5^At a first glance, this line of argumentation may appear incompatible with the fact that N400 effects are elicited even in the context of attentional blink scenarios (i.e., when a second target stimulus following in close proximity to a first target tends to be missed in terms of behavioral responses). However, in contrast to the experimental design by Chwilla et al. (1995), attential blink designs necessarily involve marking the words in question as targets by (form-based) information of some sort. For example, Rolke et al. (2001) observed an N400 priming effect even when the prime word was missed as part of an attential blink paradigm. In this study, target 1, prime and probe were presented embedded in a word list, each marked as targets by their white font color within a list of black distractor words. Thus, even though the processing of the prime word may not have “reached consciousness” as signaled by a correct target response, it was nevertheless flagged as a task-relevant stimulus by its form features (color) within the context of this experimental design. We assume that this is sufficient for some form of predictive processing to be initiated as part of a predictive coding framework.

^6^We assume that it is no accident that such non-semantic N400 effects seem to appear predominantly in languages other than English. This relates once again to the notion of precision-weighting, i.e., the dominance of word order cues for sentence interpretation in English, as a result of which these—mostly morphology-based—effects do not occur.

^7^Similar considerations hold in comparison to a range of other approaches to modeling the N400, e.g., Cheyette and Plaut (2017), who model N400 effects as “transient over-activation within semantic networks”. While these authors explicitly criticize approaches to the N400 based on implicit prediction error (e.g., Rabovsky and McRae, 2014) and therefore model N400 amplitude as directly reflecting semantic activity in a neural network, it is unclear how this model could reproduce the cross-linguistic variability in the N400 given that the semantic relations are comparable in each case.

^8^The exact functional specification of the different levels remains unknown at present. We have suggested previously that, for language processing, these could correspond approximately to linguistic units of different sizes and timescales, such as phonemes, morphemes, words, clauses etc. (Bornkessel-Schlesewsky and Schlesewsky, 2013b; Bornkessel-Schlesewsky et al., 2015). This assumption is in line with fMRI evidence (Lerner et al., 2011) and with results on the entrainment of oscillatory neural activity to different levels of linguistic categories within the speech stream (Luo and Poeppel, 2007; Ding et al., 2016).

^9^Note that we restrict our account to focal, language-related negativities. Sustained negativities such as the sustained left-anterior negativity (sLAN) likely reflect different underlying mechanisms (cf. Bornkessel-Schlesewsky and Schlesewsky, 2009a, for discussion).

## References

[B1] Alday P. M. Schlesewsky M. Bornkessel-Schlesewsky I. (2015). Discovering prominence and its role in language processing: an individual (differences) approach. Linguist. Vanguard 1, 201–213. 10.1515/lingvan-2014-1013

[B2] Alday P. M. Schlesewsky M. Bornkessel-Schlesewsky I. (2017). Electrophysiology reveals the neural dynamics of naturalistic auditory language processing: event-related potentials reflect continuous model updates. Eneuro 4, ENEURO.0311–16.2017. 10.1523/ENEURO.0311-16.2017 29379867 PMC5779117

[B3] Amsel B. D. DeLong K. A. Kutas M. (2015). Close, but no garlic: perceptuomotor and event knowledge activation during language comprehension. J. Mem. Lang. 82, 118–132. 10.1016/j.jml.2015.03.009 25897182 PMC4400663

[B4] Attneave F. (1954). Some informational aspects of visual perception. Psychol. Rev. 61, 183–193. 13167245 10.1037/h0054663

[B5] Aust F. Barth M. (2018). Papaja: Create APA Manuscripts with R Markdown. Available online at: https://github.com/crsh/papaja

[B6] Baldeweg T. Klugman A. Gruzelier J. Hirsch S. R. (2004). Mismatch negativity potentials and cognitive impairment in schizophrenia. Schizophr. Res. 69, 203–217. 10.1016/j.schres.2003.09.009 15469194

[B7] Bar M. (2003). A cortical mechanism for triggering top-down facilitation in visual object recognition. J. Cogn. Neurosci. 15, 600–609. 10.1162/089892903321662976 12803970

[B8] Bastos A. M. Usrey W. M. Adams R. A. Mangun G. R. Fries P. Friston K. J. (2012). Canonical microcircuits for predictive coding. Neuron 76, 695–711. 10.1016/j.neuron.2012.10.038 23177956 PMC3777738

[B9] Bever T. G. (1970). The cognitive basis for linguistic structures, in Cognition and the Development of Language, ed Hayes J (New York, NY: Wiley), 279–362.

[B10] Bickel B. Zakharko T. Bierkandt L. Witzlack-Makarevich A. (2014). Semantic role clustering: an empirical assessment of semantic role types in non-default case assignment. Stud. Lang. 38, 485–511. 10.5167/uzh-98913

[B11] Bornkessel I. McElree B. Schlesewsky M. Friederici A. D. (2004). Multi-dimensional contributions to garden path strength: dissociating phrase structure from case marking. J. Mem. Lang. 51, 495–522. 10.1016/j.jml.2004.06.011

[B12] Bornkessel-Schlesewsky I. Krauspenhaar S. Schlesewsky M. (2013). Yes, you can? A speaker's potency to act upon his words orchestrates early neural responses to message-level meaning. PLoS ONE 8:e69173. 10.1371/journal.pone.0069173 23894425 PMC3722173

[B13] Bornkessel-Schlesewsky I. Kretzschmar F. Tune S. Wang L. Genc S. Philipp M.. (2011). Think globally: cross-linguistic variation in electrophysiological activity during sentence comprehension. Brain Lang. 117, 133–152. 10.1016/j.bandl.2010.09.010 20970843

[B14] Bornkessel-Schlesewsky I. Schlesewsky M. (2009a). Processing Syntax and Morphology: A Neurocognitive Perspective. Oxford: Oxford University Press.

[B15] Bornkessel-Schlesewsky I. Schlesewsky M. (2009b). The role of prominence information in the real-time comprehension of transitive constructions: a cross-linguistic approach. Lang. Linguist. Compass 31, 19–58. 10.1111/j.1749-818X.2008.00099.x

[B16] Bornkessel-Schlesewsky I. Schlesewsky M. (2013a). Neurotypology: Modeling crosslinguistic similarities and differences in the neurocognition of language comprehension, in Language Down the Garden Path: The Cognitive and Biological Basis for Linguistic Structures, eds Sanz M. Laka I. Tanenhaus M. K (Oxford: Oxford University Press) 241–252.

[B17] Bornkessel-Schlesewsky I. Schlesewsky M. (2013b). Reconciling time, space and function: a new dorsal–Ventral stream model of sentence comprehension. Brain Lang. 125, 60–76. 10.1016/j.bandl.2013.01.010 23454075

[B18] Bornkessel-Schlesewsky I. Schlesewsky M. Small S. L. Rauschecker J. P. (2015). Neurobiological roots of language in primate audition: common computational properties. Trends Cogn. Sci. 19, 1–9. 10.1016/j.tics.2014.12.008 25600585 PMC4348204

[B19] Bornkessel-Schlesewsky I. Staub A. Schlesewsky M. (2016). The Timecourse of Sentence Processing in the Brain, in Neurobiology of Language, eds Hickok G. Small S. L (Amsterdam: Elsevier), 607–620. Available online at: http://linkinghub.elsevier.com/retrieve/pii/B9780124077942000493

[B20] Borovsky A. Elman J. L. Kutas M. (2012). Once is enough: N400 indexes semantic integration of novel word meanings from a single exposure in context. Lang. Learn. Dev. 8, 278–302. 10.1080/15475441.2011.614893 23125559 PMC3484686

[B21] Bourguignon N. Drury J. Valois D. Steinhauer K. (2012). Decomposing animacy reversals between agents and experiencers: an ERP study. Brain Lang. 122, 179–189. 10.1016/j.bandl.2012.05.001 22694997

[B22] Brennan J. Nir Y. Hasson U. Malach R. Heeger D. J. Pylkknen L. (2012). Syntactic structure building in the anterior temporal lobe during natural story listening. Brain Lang. 120, 163–173. 10.1016/j.bandl.2010.04.002 20472279 PMC2947556

[B23] Brilmayer I. Werner A. Primus B. Bornkessel-Schlesewsky I. Schlesewsky M. (2018). The exceptional nature of the first person in natural story processing and the transfer of egocentricity. Lang. Cogn. Neurosci. 1–17. 10.1080/23273798.2018.1542501

[B24] Brouwer H. Fitz H. Hoeks J. C. (2012). Getting real about semantic illusions: rethinking the functional role of the P600 in language comprehension. Brain Res. 1446, 127–143. 10.1016/j.brainres.2012.01.055 22361114

[B25] Cheyette S. J. Plaut D. C. (2017). Modeling the N400 ERP component as transient semantic over-activation within a neural network model of word comprehension. Cognition 162, 153–166. 10.1016/j.cognition.2016.10.016 27871623 PMC5362283

[B26] Chow W.-Y. Lau E. Wang S. Phillips C. (2018). Wait a second! Delayed impact of argument roles on on-line verb prediction. Lang. Cogn. Neurosci. 33, 803–828. 10.1080/23273798.2018.1427878

[B27] Chow W.-Y. Momma S. Smith C. Lau E. Phillips C. (2016a). Prediction as memory retrieval: timing and mechanisms. Lang. Cogn. Neurosci. 31, 617–627. 10.1080/23273798.2016.1160135

[B28] Chow W.-Y. Smith C. Lau E. Phillips C. (2016b). A “bag-of-arguments” mechanism for initial verb predictions. Lang. Cogn. Neurosci. 31, 577–596. 10.1080/23273798.2015.1066832

[B29] Christmann C. A. Berti S. Steinbrink C. Lachmann T. (2014). Differences in sensory processing of German vowels and physically matched non-speech sounds as revealed by the mismatch negativity (MMN) of the human event-related brain potential (ERP). Brain Lang. 136, 8–18. 10.1016/j.bandl.2014.07.004 25108306

[B30] Chwilla D. J. Brown C. M. Hagoort P. (1995). The N400 as a function of the level of processing. Psychophysiology 32, 274–285. 10.1111/j.1469-8986.1995.tb02956.x 7784536

[B31] Coulson S. King J. Kutas M. (1998). ERPs and Domain Specificity: Beating a Straw Horse. Lang. Cogn. Process. 13, 653–672.

[B32] Delaney-Busch N. Kuperberg G. R. (2013). Friendly drug-dealers and terrifying puppies: affective primacy can attenuate the N400 effect in emotional discourse contexts. Cogn. Affect. Behav. Neurosci. 13, 473–490. 10.3758/s13415-013-0159-5 23559312 PMC3778123

[B33] DeLong K. A. Chan W. Kutas M. (2018). Similar time courses for word form and meaning preactivation during sentence comprehension. Psychophysiology e13312. 10.1111/psyp.13312 30548266 PMC6402973

[B34] DeLong K. A. Troyer M. Kutas M. (2014). Pre-processing in sentence comprehension: sensitivity to likely upcoming meaning and structure: pre-processing in sentence comprehension. Lang. Linguist. Compass 8, 631–645. 10.1111/lnc3.12093 27525035 PMC4982702

[B35] DeLong K. A. Urbach T. P. Kutas M. (2005). Probabilistic word pre-activation during language comprehension inferred from electrical brain activity. Nat. Neurosci. 8, 1117–1121. 10.1038/nn1504 16007080

[B36] Dikker S. Pylkkänen L. (2011). Before the N400: effects of lexical–Semantic violations in visual cortex. Brain Lang. 118, 23–28. 10.1016/j.bandl.2011.02.006 21458057

[B37] Dikker S. Rabagliati H. Farmer T. A. Pylkknen L. (2010). Early occipital sensitivity to syntactic category is based on form typicality. Psychol. Sci. 21, 629–634. 10.1177/0956797610367751 20483838

[B38] Dikker S. Rabagliati H. Pylkknen L. (2009). Sensitivity to syntax in visual cortex. Cognition 110, 293–321. 10.1016/j.cognition.2008.09.008 19121826 PMC2709501

[B39] Ding N. Melloni L. Zhang H. Tian X. Poeppel D. (2016). Cortical tracking of hierarchical linguistic structures in connected speech. Nat. Neurosci. 19, 158–164. 10.1038/nn.4186 26642090 PMC4809195

[B40] Donchin E. Coles M. (1988). Is the P300 component a manifestation of context updating? Behav. Brain Sci. 11, 357–374.

[B41] Federmeier K. D. (2007). Thinking ahead: the role and roots of prediction in language comprehension. Psychophysiology 44, 491–505. 10.1111/j.1469-8986.2007.00531.x 17521377 PMC2712632

[B42] Federmeier K. D. Kutas M. (1999). A rose by any other name: long-term memory structure and sentence processing. J. Mem. Lang. 41, 469–495.

[B43] Feldman H. Friston K. J. (2010). Attention, uncertainty, and free-energy. Front. Hum. Neurosci. 4:215. 10.3389/fnhum.2010.00215 21160551 PMC3001758

[B44] Felleman D. Van Essen D. (1991). Distributed hierarchical processing in the primate cerebral cortex. Cereb. Cortex 1, 1–47. 1822724 10.1093/cercor/1.1.1-a

[B45] Frank S. L. Otten L. J. Galli G. Vigliocco G. (2015). The ERP response to the amount of information conveyed by words in sentences. Brain Lang. 140, 1–11. 10.1016/j.bandl.2014.10.006 25461915

[B46] Frenzel S. Schlesewsky M. Bornkessel-Schlesewsky I. (2015). Two routes to actorhood: lexicalized potency to act and identification of the actor role. Front. Psychol. 6:1. 10.3389/fpsyg.2015.00001 25688217 PMC4311632

[B47] Friederici A. D. (2002). Towards a neural basis of auditory sentence processing. Trends Cogn. Sci. 6, 78–84. 10.1016/S1364-6613(00)01839-8 15866191

[B48] Frisch S. Schlesewsky M. (2001). The N400 indicates problems of thematic hierarchizing. Neuroreport 12, 3391–3394. 11711892 10.1097/00001756-200110290-00048

[B49] Friston K. J. (2005). A theory of cortical responses. Philos. Trans. R. Soc. B Biol. Sci. 360, 815–836. 10.1098/rstb.2005.1622 15937014 PMC1569488

[B50] Friston K. J. (2010). The free-energy principle: a unified brain theory? Nat. Rev. Neurosci. 11, 127–138. 10.1038/nrn2787 20068583

[B51] Friston K. J. FitzGerald T. Rigoli F. Schwartenbeck P. O'Doherty J. Pezzulo G. (2016). Active inference and learning. Neurosci. Biobehav. Rev. 68, 862–879. 10.1016/j.neubiorev.2016.06.022 27375276 PMC5167251

[B52] Garrido M. I. Kilner J. M. Kiebel S. J. Stephan K. E. Friston K. J. (2007). Dynamic causal modelling of evoked potentials: a reproducibility study. Neuroimage 36, 571–580. 10.1016/j.neuroimage.2007.03.014 17478106 PMC2640482

[B53] Garrido M. I. Kilner J. M. Stephan K. E. Friston K. J. (2009). The mismatch negativity: a review of underlying mechanisms. Clin. Neurophysiol. 120, 453–463. 10.1016/j.clinph.2008.11.029 19181570 PMC2671031

[B54] Grush R. (2004). The emulation theory of representation: motor control, imagery, and perception. Behav. Brain Sci. 27, 377–396. 10.1017/S0140525X04000093 15736871

[B55] Gunter T. C. Stowe L. A. Mulder G. (1997). When syntax meets semantics. Psychophysiology 34, 660–676. 9401421 10.1111/j.1469-8986.1997.tb02142.x

[B56] Hagoort P. (2005). On Broca, brain, and binding: a new framework. Trends Cogn. Sci. 9, 416–423. 10.1016/j.tics.2005.07.004 16054419

[B57] Hagoort P. van Berkum J. J. (2007). Beyond the sentence given. Philos. Trans. R. Soc. B 362, 801–811. 10.1098/rstb.2007.2089 17412680 PMC2429998

[B58] Hahne A. Friederici A. D. (2002). Differential task effects on semantic and syntactic processes as revealed by ERPs. Cogn. Brain Res. 13, 339–356. 10.1016/S0926-6410(01)00127-6 11918999

[B59] Hasson U. Yang E. Vallines I. Heeger D. J. Rubin N. (2008). A hierarchy of temporal receptive windows in human cortex. J. Neurosci. 28, 2539–2550. 10.1523/JNEUROSCI.5487-07.2008 18322098 PMC2556707

[B60] Hasting A. S. Kotz S. A. Friederici A. D. (2007). Setting the stage for automatic syntax processing: the mismatch negativity as an indictor of syntactic priming. J. Cogn. Neurosci. 19, 386–400. 10.1162/jocn.2007.19.3.386 17335388

[B61] Haupt F. S. Schlesewsky M. Roehm D. Friederici A. D. Bornkessel-Schlesewsky I. (2008). The status of subject–object reanalyses in the language comprehension architecture. J. Mem. Lang. 59, 54–96. 10.1016/j.jml.2008.02.003

[B62] Heilbron M. Chait M. (2017). Great expectations: Is there evidence for predictive coding in auditory cortex? Neuroscience 389, 54–73. 10.1016/j.neuroscience.2017.07.061 28782642

[B63] Hohwy J. (2013). The Predictive Mind. Oxford: Oxford University Press.

[B64] Hörberg T. Koptjevskaja-Tamm M. Kallioinen P. (2013). The neurophysiological correlate to grammatical function reanalysis in Swedish. Lang. Cogn. Process. 28, 388–416. 10.1080/01690965.2011.651345

[B65] Hosemann J. Herrmann A. Steinbach M. Bornkessel-Schlesewsky I. Schlesewsky M. (2013). Lexical prediction via forward models: N400 evidence from German Sign Language. Neuropsychologia 51, 2224–2237. 10.1016/j.neuropsychologia.2013.07.013 23896445

[B66] Ito A. Corley M. Pickering M. J. Martin A. E. Nieuwland M. S. (2016). Predicting form and meaning: evidence from brain potentials. J. Mem. Lang. 86, 157–171. 10.1016/j.jml.2015.10.007

[B67] Jouravlev O. Schwartz R. Ayyash D. Mineroff Z. Gibson E. Fedorenko E. (2019). Tracking colisteners' knowledge states during language comprehension. Psychol. Sci. 30, 3–19. 10.1177/0956797618807674 30444681 PMC6344950

[B68] Kandylaki K. D. Nagels A. Tune S. Kircher T. Wiese R. Schlesewsky M. (2016). Predicting “When” in discourse engages the human dorsal auditory stream: an fMRI study using naturalistic stories. J. Neurosci. 36, 12180–12191. 10.1523/JNEUROSCI.4100-15.2016 27903727 PMC5148219

[B69] Kim A. Osterhout L. (2005). The independence of combinatory semantic processing: evidence from event-related potentials. J. Mem. Lang. 52, 205–225. 10.1016/j.jml.2004.10.002

[B70] Kok P. Rahnev D. Jehee J. F. M. Lau H. C. de Lange F. P. (2012). Attention reverses the effect of prediction in silencing sensory signals. Cereb. Cortex 22, 2197–2206. 10.1093/cercor/bhr310 22047964

[B71] Kolk H. H. Chwilla D. J. van Herten M. Oor P. (2003). Structure and limited capacity in verbal working memory: a study with event-related potentials. Brain Lang. 85, 1–36. 10.1016/S0093-934X(02)00548-5 12681346

[B72] Koster-Hale J. Saxe R. (2013). Theory of mind: a Neural prediction problem. Neuron 79, 836–848. 10.1016/j.neuron.2013.08.020 24012000 PMC4041537

[B73] Kotchoubey B. (2006). Event-related potentials, cognition, and behavior: a biological approach. Neurosci. Biobehav. Rev. 30, 42–65. 10.1016/j.neubiorev.2005.04.002 16033699

[B74] Kravitz D. Saleem K. Baker C. Ungerleider L. Mishkin M. (2013). The ventral visual pathway: an expanded neural framework for the processing of object quality. Trends Cogn. Sci. 17, 26–49. 10.1016/j.tics.2012.10.011 23265839 PMC3532569

[B75] Kuperberg G. R. (2016). Separate streams or probabilistic inference? What the N400 can tell us about the comprehension of events. Lang. Cogn. Neurosci. 31, 602–616. 10.1080/23273798.2015.1130233 27570786 PMC4996121

[B76] Kuperberg G. R. Jaeger T. F. (2016). What do we mean by prediction in language comprehension? Lang. Cogn. Neurosci. 31, 32–59. 10.1080/23273798.2015.1102299 27135040 PMC4850025

[B77] Kutas M. Federmeier K. D. (2000). Electrophysiology reveals semantic memory use in language comprehension. Trends Cogn. Sci. 4, 463–469. 10.1016/S1364-6613(00)01560-6 11115760

[B78] Kutas M. Federmeier K. D. (2011). Thirty years and counting : finding meaning in the N400 component of the event-related brain potential (ERP). Annu. Rev. Psychol. 62, 621–647. 10.1146/annurev.psych.093008.131123 20809790 PMC4052444

[B79] Kutas M. Hillyard S. A. (1984). Brain potentials during reading reflect word expectancy and semantic association. Nature 307, 161–163. 6690995 10.1038/307161a0

[B80] Laszlo S. Federmeier K. D. (2009). A beautiful day in the neighborhood: an event-related potential study of lexical relationships and prediction in context. J. Mem. Lang. 61, 326–338. 10.1016/j.jml.2009.06.004 20161064 PMC2747758

[B81] Laszlo S. Federmeier K. D. (2011). The N400 as a snapshot of interactive processing: evidence from regression analyses of orthographic neighbor and lexical associate effects. Psychophysiology 48, 176–186. 10.1111/j.1469-8986.2010.01058.x 20624252 PMC2955840

[B82] Lau E. Phillips C. Poeppel D. (2008). A cortical network for semantics: (de)constructing the N400. Nat. Rev. Neurosci. 9, 920–933. 10.1038/nrn2532 19020511

[B83] Lerner Y. Honey C. J. Silbert L. J. Hasson U. (2011). Topographic mapping of a hierarchy of temporal receptive windows using a narrated story. J. Neurosci. 31, 2906–2915. 10.1523/JNEUROSCI.3684-10.2011 21414912 PMC3089381

[B84] Levy R. (2008). Expectation-based syntactic comprehension. Cognition 106, 1126–1177. 10.1016/j.cognition.2007.05.006 17662975

[B85] Li P. Bates E. MacWhinney B. (1993). Processing a language without inflections: a reaction time study of sentence interpretation in Chinese. J. Mem. Lang. 32, 169–192.

[B86] Lotze N. Tune S. Schlesewsky M. Bornkessel-Schlesewsky I. (2011). Meaningful physical changes mediate lexical-semantic integration: top-down and form-based bottom-up information sources interact in the N400. Neuropsychologia 49, 3573–3582. 10.1016/j.neuropsychologia.2011.09.009 21939678

[B87] Luo H. Poeppel D. (2007). Phase patterns of neuronal responses reliably discriminate speech in human auditory cortex. Neuron 54, 1001–1010. 10.1016/j.neuron.2007.06.004 17582338 PMC2703451

[B88] MacWhinney B. Bates E. Kliegl R. (1984). Cue validity and sentence interpretation in English, German and Italian. J. Verbal Learn. Verbal Behav. 23, 127–150.

[B89] Maier A. Adams G. K. Aura C. Leopold D. A. (2010). Distinct superficial and deep laminar domains of activity in the visual cortex during rest and stimulation. Front. Syst. Neurosci. 4:31. 10.3389/fnsys.2010.00031 20802856 PMC2928665

[B90] Menning H. Zwitserlood P. Schöning S. Hihn H. Bölte J. Dobel C.. (2005). Pre-attentive detection of syntactic and semantic errors. Neuroreport 16, 77–80. 15618895 10.1097/00001756-200501190-00018

[B91] Metusalem R. Kutas M. Urbach T. P. Hare M. McRae K. Elman J. L. (2012). Generalized event knowledge activation during online sentence comprehension. J. Mem. Lang. 66, 545–567. 10.1016/j.jml.2012.01.001 22711976 PMC3375826

[B92] Moran R. J. Symmonds M. Dolan R. J. Friston K. J. (2014). The brain ages optimally to model its environment: evidence from sensory learning over the adult lifespan. PLoS Comput. Biol. 10:e1003422. 10.1371/journal.pcbi.1003422 24465195 PMC3900375

[B93] Näätänen R. Paavilainen P. Rinne T. Alho K. (2007). The mismatch negativity (MMN) in basic research of central auditory processing: a review. Clin. Neurophysiol. 118, 2544–2590. 10.1016/j.clinph.2007.04.026 17931964

[B94] Neville H. Nicol J. Barss A. Forster K. Garret M. (1991). Syntactically based sentence processing classes: evidence from event-related brain potentials. J. Cogn. Neurosci. 3, 151–165. 23972090 10.1162/jocn.1991.3.2.151

[B95] Nevins A. Dillon B. Malhotra S. Phillips C. (2007). The role of feature-number and feature-type in processing Hindi verb agreement violations. Brain Res. 1164, 81–94. 10.1016/j.brainres.2007.05.058 17658491

[B96] Nieuwenhuis S. Aston-Jones G. Cohen J. D. (2005). Decision making, the P3, and the locus coeruleus-norepinephrine system. Psychol. Bull. 131, 510–532. 10.1037/0033-2909.131.4.510 16060800

[B97] Nieuwland M. S. (2019). Do “early” brain responses reveal word form prediction during language comprehension? A critical review. Neurosci. Biobehav. Rev. 96, 367–400. 10.1016/j.neubiorev.2018.11.019 30621862

[B98] Nieuwland M. S. Politzer-Ahles S. Heyselaar E. Segaert K. Darley E. Kazanina N.. (2018). Large-scale replication study reveals a limit on probabilistic prediction in language comprehension. eLife 7:e33468. 10.7554/eLife.33468 29631695 PMC5896878

[B99] Osterhout L. Nicol J. (1999). On the distinctiveness, independence, and time course of the brain response to syntactic and semantic anomalies. Lang. Cogn. Process. 14, 283–317.

[B100] Polich J. (2007). Updating P300: an integrative theory of P3a and P3b. Clin. Neurophysiol. 118, 2128–2148. 10.1016/j.clinph.2007.04.019 17573239 PMC2715154

[B101] Pulvermüller F. Shtyrov Y. (2003). Automatic processing of grammar in the human brain as revealed by the mismatch negativity. Neuroimage 20, 159–172. 10.1016/S1053-8119(03)00261-1 14527578

[B102] Pulvermüller F. Shtyrov Y. Hauk O. (2009). Understanding in an instant: Neurophysiological evidence for mechanistic language circuits in the brain. Brain Lang. 110, 81–94. 10.1016/j.bandl.2008.12.001 19664815 PMC2734884

[B103] Rabovsky M. Hansen S. S. McClelland J. L. (2018). Modelling the N400 brain potential as change in a probabilistic representation of meaning. Nat. Hum. Behav. 2, 693–705. 10.1038/s41562-018-0406-4 31346278

[B104] Rabovsky M. McRae K. (2014). Simulating the N400 ERP component as semantic network error: insights from a feature-based connectionist attractor model of word meaning. Cognition 132, 68–89. 10.1016/j.cognition.2014.03.010 24762924

[B105] Rao R. P. Ballard D. H. (1999). Predictive coding in the visual cortex: a functional interpretation of some extra-classical receptive-field effects. Nat. Neurosci. 2, 79–87. 10195184 10.1038/4580

[B106] Rolke B. Heil M. Streb J. Hennighausen E. (2001). Missed prime words within the attentional blink evoke an N400 semantic priming effect. Psychophysiology 38, 165–174. 11347861

[B107] Rueschemeyer S.-A. Gardner T. Stoner C. (2015). The Social N400 effect: how the presence of other listeners affects language comprehension. Psychon. Bull. Rev. 22, 128–134. 10.3758/s13423-014-0654-x 24825307

[B108] Sams M. Paavilainen P. Alho K. Näätänen R. (1985). Auditory frequency discrimination and event-related potentials. Clin. Neurophysiol. 62, 437–448. 10.1016/0168-5597(85)90054-1 2415340

[B109] Sassenhagen J. Bornkessel-Schlesewsky I. (2015). The P600 as a correlate of ventral attention network reorientation. Cortex 66, A3–A20. 10.1016/j.cortex.2014.12.019 25791606

[B110] Sassenhagen J. Schlesewsky M. Bornkessel-Schlesewsky I. (2014). The P600-as-P3 hypothesis revisited: single-trial analyses reveal that the late EEG positivity following linguistically deviant material is reaction time aligned. Brain Lang. 137, 29–39. 10.1016/j.bandl.2014.07.010 25151545

[B111] Shtyrov Y. Pulvermüller F. (2002). Memory traces for inflectional affixes as shown by mismatch negativity. Eur. J. Neurosci. 15, 1085–1091. 10.1046/j.1460-9568.2002.01941.x 11918667

[B112] Shtyrov Y. Pulvermüller F. (2007). Early MEG activation dynamics in the left temporal and inferior frontal cortex reflect semantic context integration. J. Cogn. Neurosci. 19, 1633–1642. 10.1162/jocn.2007.19.10.1633 17854281

[B113] Simon H. A. (1962). The architecture of complexity. Proc. Am. Philos. Soc. 106, 467–482.

[B114] Small S. L. (2008). The neuroscience of language. Brain Lang. 106, 1–3. 10.1016/j.bandl.2008.05.004 18544446 PMC2464569

[B115] Small S. L. Hickok G. Nusbaum H. C. Blumstein S. Branch Coslett H. Dell G. (2011). The neurobiology of language: two years later. Brain Lang. 116, 103–104. 10.1016/j.bandl.2011.02.004

[B116] Steinhauer K. Drury J. (2012). On the early left-anterior negativity (ELAN) in syntax studies. Brain Lang. 120, 135–162. 10.1016/j.bandl.2011.07.001 21924483

[B117] Stroud C. Phillips C. (2012). Examining the evidence for an independent semantic analyzer: an ERP study in Spanish. Brain Lang. 120, 108–126. 10.1016/j.bandl.2011.02.001 21377198

[B118] Szewczyk J. M. Schriefers H. (2013). Prediction in language comprehension beyond specific words: an ERP study on sentence comprehension in Polish. J. Mem. Lang. 68, 297–314. 10.1016/j.jml.2012.12.002

[B119] Todd J. Heathcote A. Mullens D. Whitson L. R. Provost A. Winkler I. (2014). What controls gain in gain control? Mismatch negativity (MMN), priors and system biases. Brain Topogr. 27, 578–589. 10.1007/s10548-013-0344-4 24343248

[B120] Troyer M. Kutas M. (2018). Harry Potter and the Chamber of *What* ?: the impact of what individuals know on word processing during reading. Lang. Cogn. Neurosci., 1–17. 10.1080/23273798.2018.1503309 PMC7531766 33015219

[B121] Tune S. Schlesewsky M. Small S. L. Sanford A. J. Bohan J. Sassenhagen J.. (2014). Cross-linguistic variation in the neurophysiological response to semantic processing: evidence from anomalies at the borderline of awareness. Neuropsychologia 56, 147–166. 10.1016/j.neuropsychologia.2014.01.007 24447768 PMC3966966

[B122] van de Meerendonk N. Kolk H. H. Chwilla D. J. Vissers C. T. W. M. (2009). Monitoring in language perception. Lang. Linguist. Compass 3, 1211–1224. 10.1111/j.1749-818X.2009.00163.x

[B123] Van Petten C. Kutas M. (1990). Interactions between sentence context and word frequencyinevent-related brainpotentials. Mem. Cogn. 18, 380–393. 2381317 10.3758/bf03197127

[B124] Van Petten C. Luka B. (2012). Prediction during language comprehension: benefits, costs, and ERP components. Int. J. Psychophysiol. 83, 176–190. 10.1016/j.ijpsycho.2011.09.015 22019481

[B125] Wacongne C. Labyt E. van Wassenhove V. Bekinschtein T. Naccache L. Dehaene S. (2011). Evidence for a hierarchy of predictions and prediction errors in human cortex. Proc. Natl. Acad. Sci. U.S.A. 108, 20754–20759. 10.1073/pnas.1117807108 22147913 PMC3251061

[B126] Weiss A. F. Kretzschmar F. Schlesewsky M. Bornkessel-Schlesewsky I. Staub A. (2017). Comprehension demands modulate re-reading, but not first pass reading behavior. Q. J. Exp. Psychol., 1–37. 10.1080/17470218.2017.1307862. [Epub ahead of print]. 28300468

[B127] Wicha N. Y. Y. Moreno E. M. Kutas M. (2004). Anticipating words and their gender: an event-related brain potential study of semantic integration, gender expectancy, and gender agreement in Spanish sentence reading. J. Cogn. Neurosci. 16, 1272–1288. 10.1162/0898929041920487 15453979 PMC3380438

[B128] Zachau S. Rinker T. Körner B. Kohls G. Maas V. Hennighausen K.. (2005). Extracting rules: early and late mismatch negativity to tone patterns. NeuroReport 16, 2015–2019. 16317345 10.1097/00001756-200512190-00009

